# Concentrations of oocyte secreted GDF9 and BMP15 decrease with MII transition during human IVM

**DOI:** 10.1186/s12958-022-01000-6

**Published:** 2022-08-19

**Authors:** Jesús Cadenas, Susanne Elisabeth Pors, Ajay Kumar, Bhanu Kalra, Stine Gry Kristensen, Claus Yding Andersen, Linn Salto Mamsen

**Affiliations:** 1 Laboratory of Reproductive Biology, The Juliane Marie Centre for Women, Children and Reproduction, University Hospital of Copenhagen, Rigshospitalet, 2100 Copenhagen, Denmark; 2 Ansh Labs LLC, 445 W. Medical Center Blvd, Webster, TX 77598 USA

**Keywords:** GDF9, BMP15, GDF9, BMP15 heterodimer, Cumulin, Ex-vivo collected oocytes, Human oocyte maturation

## Abstract

**Background:**

The suggested effects of the oocyte secreted GDF9 and BMP15 growth factors on oocyte maturation are currently based on recombinant proteins, and little is known about native GDF9 and BMP15 in humans.

**Methods:**

Human immature cumulus-oocyte complexes (COCs) obtained in connection with ovarian tissue cryopreservation (OTC) underwent in vitro maturation (IVM). Oocyte-produced GDF9 and BMP15 were detected in COCs using immunofluorescence, and in fresh GV oocytes and in GV and MII oocytes after IVM by western blot. Concentrations of GDF9, BMP15 homodimers, and GDF9/BMP15 heterodimer in spent media after IVM were measured by ELISA. The relative expression of seven genes from the GDF9 and BMP15 signaling pathways (*BMPR2, ALK5, ALK6, SMAD1, SMAD2, SMAD3,* and *SMAD5*) was evaluated in fresh cumulus cells (before IVM) and in cumulus cells from GV and MII oocytes after IVM by RT-qPCR.

**Results:**

We detected native pro-mature GDF9 and BMP15 in human oocytes with molecular weights (Mw) of 47 kDa and 43 kDa, respectively. Concentrations of GDF9 and BMP15 in spent media after IVM were detected in 99% and 64% of the samples, respectively. The GDF9/BMP15 heterodimer was detected in 76% of the samples. Overall, the concentration of GDF9 was approximately 10-times higher than BMP15. The concentrations of both GDF9 and BMP15 were significantly lower in spent medium from MII oocytes than in media from oocytes that remained at the GV stage. Concentrations of the GDF9/BMP15 heterodimer did not differ between GV and MII oocytes. Furthermore, *BMPR2*, *SMAD3*, and *SMAD5* were significantly upregulated in cumulus cells from MII oocytes, indicating that both GDF9 and BMP15 signaling were active during oocyte meiotic resumption in vitro*.*

**Conclusion:**

These data suggest that the driving mechanisms for oocyte nuclear maturation may involve both GDF9 and BMP15 homodimers, while the role of the GDF9/BMP15 heterodimer is questionable.

**Supplementary Information:**

The online version contains supplementary material available at 10.1186/s12958-022-01000-6.

## Introduction

Oocyte growth and maturation in vivo is regulated by endocrine, paracrine, and autocrine control systems that are crucial for acquiring oocyte competence [1–3]. The oocyte itself plays a major role in the regulation of follicle development and modulates its microenvironment by secreting growth factors that signal through the transforming growth factor-beta (TGF-β) signaling pathways [4], such as growth and differentiation factor 9 (GDF9) and bone morphogenetic protein 15 (BMP15). Both these growth factors are secreted solely by the oocyte to affect granulosa and cumulus cells, with downstream effects on follicle development, oocyte maturation, and ovulation [1, 4].

Knowledge on the involvement of GDF9 and BMP15 in oocyte maturation derives from studies using animal models and/or recombinant proteins (rGDF9 and rBMP15). Thus, oocyte competence in vitro, but not meiotic maturation, was improved when rGDF9 and/or rBMP15 were added to culture medium in several mammalian species [5–8]. Both GDF9 and BMP15 are synthesized as precursor proteins with pro- and mature domains that are further processed to form homodimers or heterodimers that bind to specific serine-threonine transmembrane receptors in granulosa and cumulus cells [9]. Mature GDF9 binds to BMP receptor type II (BMPR2), which then recruits the activin receptor-like kinase-5 (ALK5) and activates the SMAD2/3 pathway [10, 11]. Mature BMP15, on the other hand, binds to complexes of BMPR2 and ALK6 activating the SMAD1/5/8 pathway [12].

The molecular weights (Mw) of pro-mature GDF9 and BMP15 in human follicle fluids are suggested to be around 50 kDa and the mature forms around 20 kDa [13], and 60 and 20 kDa in HEK cell lines, respectively [8]. Though the molecular forms of human GDF9 and BMP15 within the oocyte remain unclear. Human GDF9 is secreted in a pro-mature form that must be processed to become bioactive, unlike in mice, in which GDF9 is secreted in an active form [8]. Further, in vitro studies in HEK cell lines have shown that BMP15 is produced in an active form in humans, whereas ovine BMP15 is inactive, and mouse BMP15 is misfolded and not secreted from the cells [14], indicating different regulation of these factors between species.

A study with GDF9 and BMP15 double knockout mice showed a synergistic biological action of GDF9 and BMP15 [15]. However, whether this cooperation results from the interaction between GDF9 and BMP15 homodimers or from a GDF9/BMP15 heterodimer is still a matter of debate. There is a body of evidence pointing towards species-specific differences in the GDF9/BMP15 system in vivo [14, 16, 17]. For instance, GDF9-deficient mice are infertile [18], and BMP15-deficient mice are subfertile [15], whereas in sheep both proteins are required for the animal to be fertile [19, 20]. In humans, dysregulation or naturally occurring mutations within the GDF9/BMP15 system have been linked to several female reproductive diseases, including primary ovarian insufficiency and polycystic ovarian syndrome [3]. However, reports are not consistent [21–29]. These differences between species suggest that effects observed in mutant and knock-out animal models may not reflect mechanisms in humans.

An increased understanding of BMP15/GDF9 signaling during human oocyte maturation may improve the clinical treatment for women with ovulation dysfunction and infertility, especially in connection with in vitro maturation (IVM). Also, levels of GDF9 and BMP15 may serve as biomarkers for oocyte quality. To date, quantitation of these proteins has proven difficult due to several factors: (i) the lack of specific monoclonal antibodies available, needed for assay development; (ii) BMP15 and GDF9 have some atypical structural features (e.g., they form non-covalently homo- and heterodimers); (iii) it is unknown exactly how GDF9 and BMP15 are processed in vivo and which forms are present in human biological fluids. Only a few studies have quantitated GDF9 and BMP15 in human serum and follicle fluids using either in-house developed enzyme-linked immunosorbent assays (ELISA) or a new commercially available ELISA for GDF9 [29–32].The present study aimed to characterize human native GDF9 and BMP15 and explore their regulation during IVM using western blot, immunofluorescence, and ELISA analyses in human oocytes and spent media after IVM. Moreover, the expression of genes down-stream of GDF9 and BMP15 signaling was also evaluated in cumulus cells before and after IVM.

## Materials and methods

### Patients and collected material

A total of 60 patients (mean age 28 years; range 13–39) who underwent unilateral ovariectomy and ovarian tissue cryopreservation were included in the study. The indications for fertility preservation were breast cancer (*n* = 39), neurological malignancy (*n* = 5), non-malignant blood disorders (*n* = 5), lymphoma (*n* = 4), gastrointestinal malignancy (*n* = 3), other malignant diseases (*n* = 3), and other benign disease (*n* = 1).. All samples used in the present study (oocytes, cumulus cells, and spent media) were collected in association with other research projects. The type of sample used from each patient is shown in Additional file 1: Table S1. Information on the number of oocytes collected per patient and IVM outcomes have been previously published [33, 34].

### Ovary transport and oocyte collection

After ovariectomy, ovaries were transported in IVF flushing medium (Origio A/S, Måløv, Denmark) either at 37 °C from the local hospital (10 min transport) or on crushed ice from collaborating hospitals (2–5 h transport). Once the ovarian cortex was isolated for cryopreservation, all dishes containing the surplus medulla tissue in HEPES-buffered HTF medium (Invitrogen, GIBCO™) were examined for the presence of immature oocytes under a stereomicroscope (Leica MZ12, Germany) in a flow hood with heated tabletop at 37 °C. Recovered oocytes were placed in holding medium as previously described [33]. Only oocytes that showed clear signs of degeneration, such as darkened cytoplasm, were excluded from the study. Fresh oocytes for western blot analysis were snap frozen right after collection and stored at -80℃ until analysis.

### Tissue processing and immunofluorescence staining

Isolated cumulus-oocyte complexes (COCs) for immunofluorescence analyses were embedded in 1% alginate solution followed by embedding in 4% agar solution and fixated in 10% neutral buffered formalin for 4 h, washed in graded ethanol, and embedded in paraffin. COCs were cut into 5 µm serial sections and prepared for immunofluorescence staining as described previously with few modifications including no use of antigen retrieval and a blocking step with 5% normal donkey serum (Abcam, Cambridge, UK, cat.no.: ab7475) [35]. Mouse monoclonal GDF9 or BMP15 antibodies (Ansh Labs, LLC, Webster, TX, USA, 1:100) were used together with a HSD17β1 rabbit monoclonal antibody (Abcam, Cat. No.: ab51045, 1:100). Universal negative control serum® (BioCare Medical, CA, USA) was used in place of primary antibody as negative control and showed no staining; data not shown.

### In vitro maturation of human oocytes and collection of cumulus cells and spent media

**Fig. 1:**
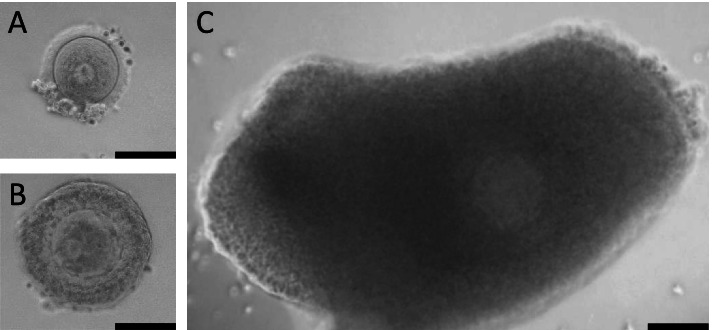
Immature oocytes collected from the surplus medulla tissue during OTC. (**A**) Naked oocyte (NO). (**B**) Cumulus-oocyte complex (COC) with small cumulus mass (S-COC). (**C**) COC with large cumulus mass (L-COC). Scale bars: 100 µm

Oocyte IVM and cumulus cell collection were performed as described previously with minor modifications [34]. Briefly, COCs were divided into three categories according to cumulus mass size: Large cumulus mass (L-COCs), small cumulus mass (S-COCs) and naked oocytes (NOs) (Fig. 1). COCs with at least 10 layers of cumulus cells were considered L-COCs. The COCs were washed three times in IVM medium which consisted of MediCult IVM system (Origio A/S, Denmark) supplemented with 10 mg/mL human serum albumin (HAS; CSL Behring 20%, Germany), 100 IU/L human rLH (Luveris, Serono, Germany), 75 IU/L human rFSH (Rekovelle, Ferring, Copenhagen, Denmark), and 1 µg/mL human recombinant Midkine (SRP3114, Sigma-Aldrich, USA), and then individually transferred to fresh 25-µl drops of IVM medium. All oocytes were incubated under paraffin oil (Origio A/S, Denmark) for 42 h at 37 °C with 5% CO_2_ in air. After 42 h, COCs were mechanically denuded with a 130–133 μm denudation pipette (Vitrolife, Gothenburg, Sweden). Under an inverted microscope (Carl Zeiss Axiovert 135, Germany; × 20 magnification), oocytes were classified as germinal vesicle (GV), metaphase I (MI), metaphase II (MII), or degenerated (DEG). Oocyte maturation was determined by the presence of the first polar body (MII oocytes). The spent media and/or cumulus cells from non-degenerated L-COCs containing GV and MII oocytes after IVM were individually collected and stored at -80 °C for further analyses.

### Determination of GDF9, BMP15, and GDF9/BMP15 heterodimer by ELISA

Concentrations of GDF9, BMP15 and GDF9/BMP15 heterodimer were measured using ELISA assays in single drops of spent media (*n* = 94) from L-COCs containing GV (*n* = 43) or MII oocytes (*n* = 51) after IVM. The measurements were performed using GDF9 (AL-176), BMP15 (AL-179), GDF9-BMP15 complex (AL-181) ELISA’s from Ansh Labs, Webster, Texas, USA. All samples were diluted 1:5 prior to assaying GDF9/BMP15 heterodimer and diluted 1:10 prior to measuring BMP15, and GDF9, respectively. Analytical characteristics for GDF9, BMP15 and the GDF9-BMP15 complex assays are described in Additional file 2: Table S2.

### Quantitative real-time PCR (RT-qPCR) analysis

Cumulus cells (*n* = 72) from COCs before (Fresh, *n* = 24) and after IVM (*n* = 48) were evaluated according to their maturation status: GV (*n* = 21) or MII (*n* = 27). Total RNA was individually extracted and purified from cumulus cells of each COC as previously described using the Agilent RNA 6000 Pico Kit and Taqman® assay [34]. The following TaqMan probes were used: BMP receptor type 2 (*BMPR2*; #Hs000176148_m1), *ALK5* (#Hs00610320_m1), *ALK6* (#Hs01010965_m1), *SMAD1* (#Hs00195432_m1), *SMAD2* (#Hs00183425_m1), *SMAD3* (#Hs00969210_m1), *SMAD5* (#Hs00195437_m1), and beta-actin (*ACTB*; #Hs01060665_g1) All samples were run in duplicates and normalized to *ACTB* [36].

### Western blot

Western blot analyses were performed as previously described [37] using the Invitrogen western blot system (Thermo Fischer, Hvidovre, Denmark). Pooled oocytes were lysed in 20 µl radioimmunoprecipitation assay (RIPA) buffer (R0278, Sigma-Aldrich, Brøndby, Denmark). Proteins separated on a NuPAGE® 4–12% Bis–Tris mine gel was transferred to a membrane blocked in 5% skim milk and incubated with primary antibody (GDF9 or BMP15, Ansh Labs, 1:5000) overnight at 4 °C. Secondary horseradish peroxidase-conjugated rabbit-anti-mouse antibody (1:1000, Sigma-Aldrich) was applied for 1 h at room temperature. The Mw of the visualized bands were calculated using the AzureSpot 2.2.170 software.

The specificity of the primary antibodies used for western blotting was tested with blocking peptides against GDF9 (BG016, Ansh Labs) and BMP15 (BB028, Ansh Labs). Human rGDF9 standard (SRP4872, Sigma Aldrich) and human rBMP15 standard (5096-BM-005, R&D systems, Abingdon, UK) were used as a positive control.

### Statistical analysis

The association between growth factor concentration in spent media and oocyte maturation was modeled as a mixed logistic regression with the concentrations of GDF9, BMP15, and GDF9/BMP15 heterodimer as outcome and oocyte stage (GV and MII) as explanatory variable. Patient effect was included as a random intercept. The impact of oocyte maturation stage on expression of the target genes (*BMPR2*, *ALK5*, *ALK6*, *SMAD1*, *SMAD2*, *SMAD3*, and *SMAD5*) was modeled as a mixed linear regression with expression of genes as outcome and maturation (MII yes/no) as explanatory variable. Furthermore, a post hoc test (Tukey) was performed to identify differences among the groups. A two-tailed Mann–Whitney test was performed to evaluate if the Mw of pro-mature GDF9 and BMP15 detected with western blot analysis were different. All analysis was done using R version 3.4.3 or GraphPad Prism 9.3.1. All *P*-values below 0.05 were regarded as significant.

## Results

### Western blot

**Fig. 2:**
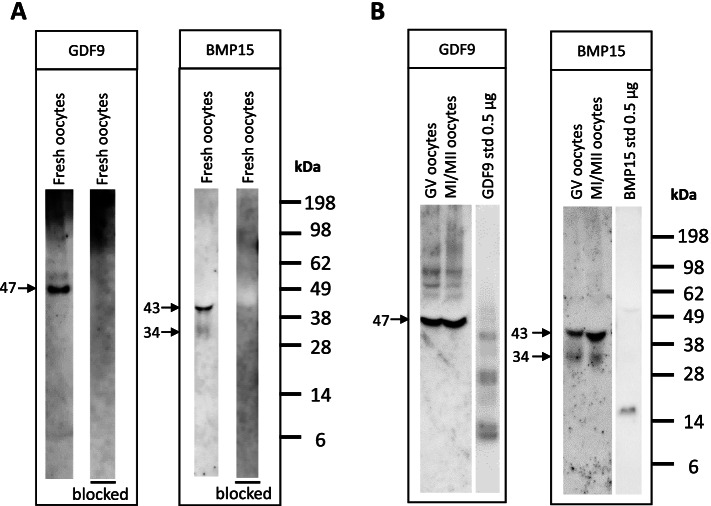
GDF9 and BMP15 in human oocytes. (**A**) GDF9 was detected at a molecular weight of 47 kDa in a pool of 11 human immature oocytes. Using a blocking peptide against GDF9 (BG016, Ansh Labs) the band disappeared. BMP15 was detected at molecular weight of 43 kDa in a pool of 20 human immature oocytes. After including a BMP15 blocking peptide (BB028, Ansh Labs) the band disappeared, validating the specificity of the antibody. **(B)** GDF9 and BMP15 were detected after IVM in pools of 23 GV oocytes per lane and 28 MI/MII oocytes per lane. Note that the bands from recombinant GDF9 and BMP15 standards differed significantly from the native oocyte derived bands. The uncropped western blot membranes can be found in additional files 3 (A) and 4 (B)

In pools of fresh GV oocytes we detected a pro-mature GDF9 band with a mean weight of 47 kDa (range: 43–49 kDa; ± SEM:1.07), and a pro-mature BMP15 band with a mean weight of 43 kDa (range: 40–45 kDa; ± SEM:1.31). Moreover, a weaker BMP15 band of 34 kDa (range: 31–37 kDa; ± SEM:1.47) were detected (one representative blot is included in Fig. 2A). The Mw of GDF9 and BMP15 were calculated as means based on 4 and 5 consecutive western blots, respectively. The Mann–Whitney test identified the pro-mature GDF9 and BMP15 bands as significantly different (*P* = 0.03). The theoretical Mw of pro-mature GDF9 and BMP15 were 48 kDa and 43 kDa, respectively, based on the sequences obtained with Blast analysis in the Uniprot database (https://www.uniprot.org/) and sequence analysis in Bioinformatics.org/sms/prot.mw.html. The primary antibody incubated over-night with either GDF9 or BMP15 blocking peptides resulted in no detectable bands, illustrating the specificity of the antibodies (Fig. 2A). The lanes presented in Fig. 2A were run on the same gel and bands from the pooled fresh oocytes served as positive controls for the lanes treated with blocking peptides. Western blot analysis was also performed using pooled GV and MI/MII oocytes after IVM and similar bands were detected (Fig. 2B), illustrating that neither IVM conditions nor oocyte meiotic resumption affected GDF9 and BMP15 production (Fig. 2B). Interestingly, the recombinant GDF9 and BMP15 standards had different Mw than the native forms of these proteins (Fig. 2B).

**Fig. 3:**
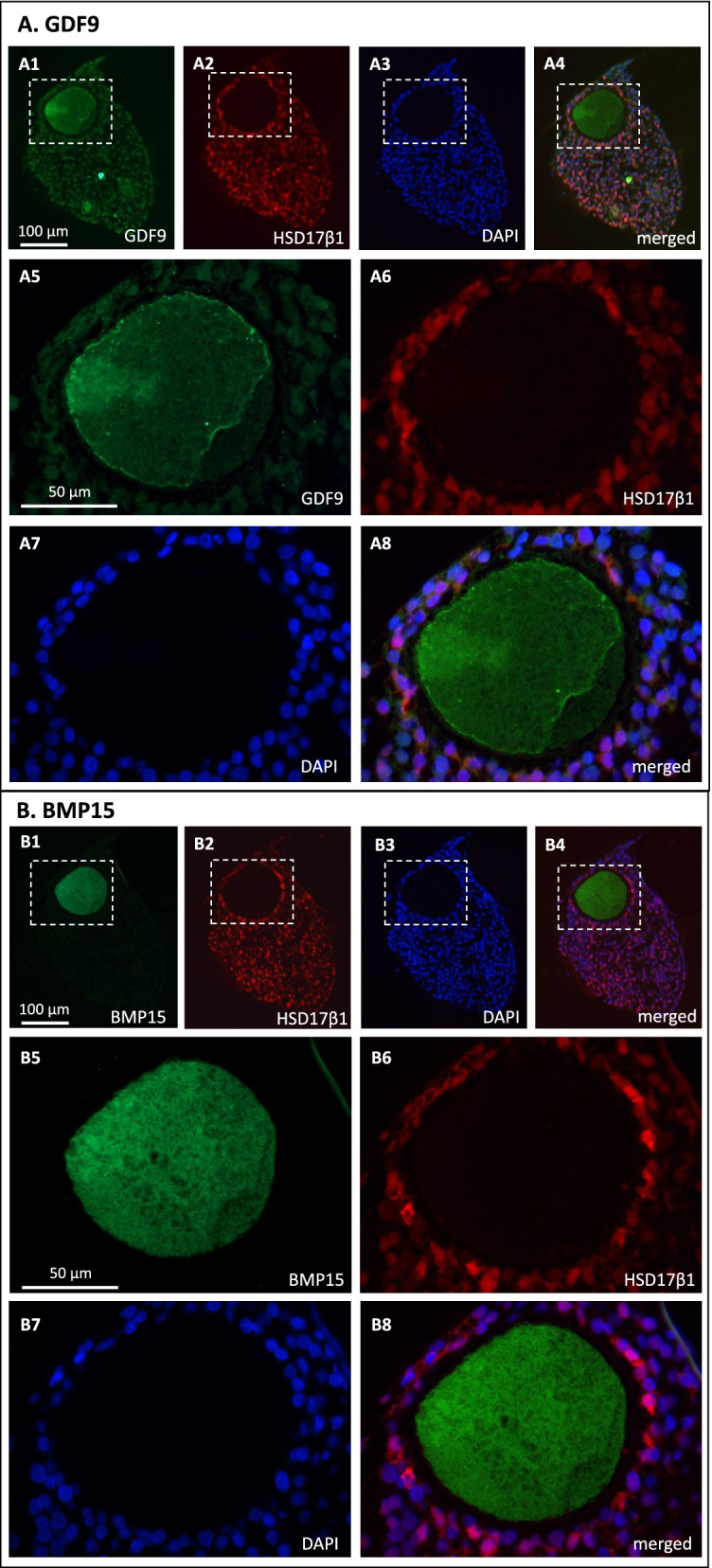
GDF9 and BMP15 localization in human ovarian follicles. (**A**) GDF9 (green) and HSD17β1 (red). (**A1-A4**) cumulus oocyte complex, (**A5-A8**) Magnifications of the dotted boxes in A1-A4). (**B**) BMP15 (green) and HSD17β1 (red). (**B1-B4**) cumulus oocyte complex, (**B5-B8**) Magnifications of the dotted boxes in B1-B4)

### Localization of GDF9 and BMP15 in human COCs

Both GDF9 and BMP15 were detected specifically in the oocyte of human COCs obtained from small antral follicles (Figs. 3 – A2 and B2). The granulosa cell specific enzyme HSD17β1 was detected in the surrounding cumulus cells (Figs. 3 – A3 and B3).

### Concentrations of GDF9, BMP15, and GDF9/BMP15 heterodimer in spent media after IVM

The concentrations of GDF9, BMP15, and the GDF9/BMP15 heterodimer were individually measured in spent media from 94 oocytes at different maturation stages after IVM: 43 at germinal vesicle (GV) and 51 at metaphase II (MII). GDF9 was detected in 93 samples: GV (*n* = 43) and MII (*n* = 50); BMP15 was detected in 60 samples: GV (*n* = 27) and MII (*n* = 33); and GDF9/BMP15 heterodimer was detected in 71 samples: GV (*n* = 31) and MII (*n* = 40).

**Fig. 4:**
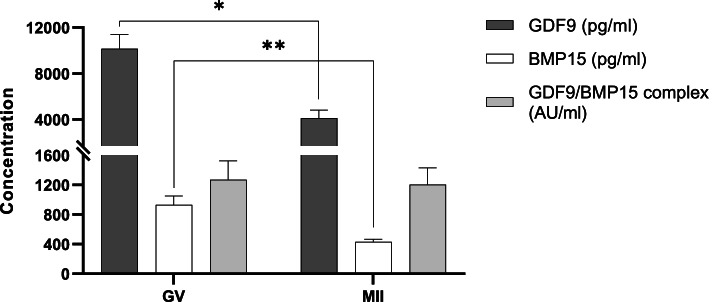
Mean concentration of GDF9, BMP15, and GDF9/BMP15 heterodimer in spent media after IVM according to oocyte meiotic stage: germinal vesicle (GV; *n* = 43) or metaphase II (MII; *n* = 51). Error bars represent SEM. *P = 0.02; **P = 0.002

Overall, the concentration of both GDF9 and BMP15 was significantly negatively associated with oocyte maturation (*P* = 0.02 and *P* = 0.004, respectively), while no significant associations were observed for the GDF9/BMP15 heterodimer (*P* > 0.05) (Fig. 4). Significant higher GDF9 and BMP15 concentrations were detected in spent media from GV oocytes compared to MII oocytes (*P* = 0.02 and *P* = 0.002, respectively) (Fig. 4). Interestingly, the GDF9/BMP15 ratio was constant within each oocyte category; while the GDF9 concentration was around ten times higher than BMP15 in spent media from both GV oocytes (10,144 pg/ml GDF9 and 931 pg/ml BMP15) and MII oocytes (4,126 pg/ml GDF9 and 430 pg/ml BMP15).

### Relative expression of BMPR2, ALK5, ALK6, SMAD1, SMAD2, SMAD3, and SMAD5 in cumulus cells

**Fig. 5:**
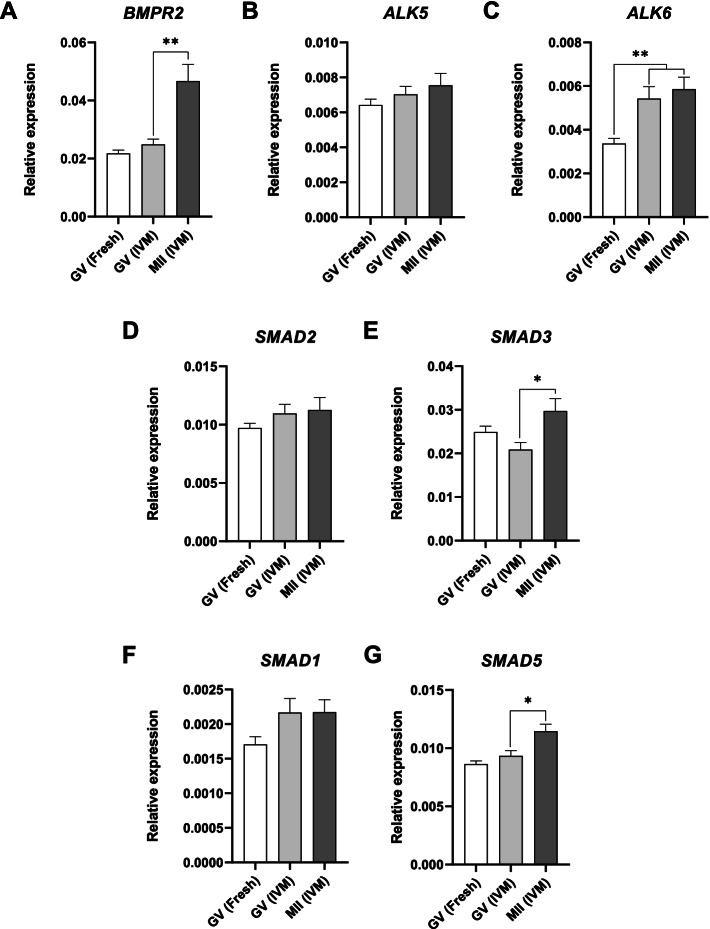
Relative expression of seven genes from the GDF9 and BMP15 signaling pathways in cumulus cells from human oocytes before IVM (GV(Fresh), *n* = 24), and after IVM (GV, *n* = 21; and MII, *n* = 27). GV and MII respectively refer to germinal vesicle and metaphase II stages. (**A**) *BMPR2*, (**B**) *ALK5*, (**C**) *ALK 6*, (**D**) *SMAD2*, (**E**) *SMAD3,* (**F**) *SMAD1*, and (**G**) *SMAD5.* Error bars represent SEM. *P < 0.05; **P ≤ 0.005

The expression of seven genes from the GDF9 and/or BMP15 signaling pathways was evaluated in cumulus cells from human oocytes before IVM (GV(Fresh), *n* = 24), and after IVM (GV, *n* = 21; and MII, *n* = 27) (Fig. 5). Overall, there was a significant positive association between oocyte maturation and the expression of *BMPR2* (*P* = 0.003), *SMAD3* (*P* = 0.02), and *SMAD5* (*P* = 0.02) in their corresponding cumulus cells (Fig. 5A,E,G). Cumulus cells from MII oocytes expressed higher levels of *BMPR2* (*P* = 0.005) (Fig. 5A), *SMAD3* (*P* = 0.02) (Fig. 5E), and *SMAD5* (*P* = 0.03) (Fig. 5G) than cumulus cells from GV oocytes after IVM. The expression of *ALK5* (Fig. 5B), *ALK6* (Fig. 5C), *SMAD1* (Fig. 5F), and *SMAD2* (Fig. 5D) did not change during oocyte meiotic resumption in vitro (*P* > 0.05). Except for *ALK6* (*P* = 0.003) (Fig. 5C), no differences were found between GV oocytes before IVM and after IVM (*P* > 0.05).

## Discussion

To our knowledge, this is the first time that native forms of human GDF9, BMP15, and the GDF9/BMP15 heterodimer have been measured in spent media after IVM and the pro-mature forms in human oocytes before and after IVM; and directly linked to oocyte meiotic maturation. We detected native pro-mature GDF9 and BMP15 in human oocytes with Mw of 47 kDa and 43 kDa, respectively, which were similar to the theoretical Mw of pro-mature GDF9 (48 kDa) and BMP15 (43 kDa). The Mw for GDF9 and BMP15 has now for the first time been detected in human oocytes in four/five independent measurements and show relative low variability. In lanes with rGDF9 and rBMP15, lower Mw bands were detected, illustrating that the antibodies were able to detect epitopes on the dissociated mature forms. Therefore, the absence of low Mw bands in the oocytes suggest that cleavage of the pro-forms does not take place inside the oocyte in humans and that dimerization takes place outside the oocyte. Thereby, these data for the first time confirm the model proposed by Stocker and co-workers [8] in humans. Further, extracellular cleavage has been reported in human follicle fluids obtained in connection with IVF [13] and supernatants from HEK cell lines [8].

Both GDF9 and BMP15 concentrations were significantly lower in spent media from MII oocytes compared to GV oocytes after IVM, whereas the GDF9/BMP15 heterodimer remained constant. However, we cannot determine whether these values of the heterodimer are high or low since we can only measure this in relative concentrations. The synergistic biological action of GDF9 and BMP15 has been widely debated [3, 15]. Nevertheless, it is still unclear whether this cooperation results from the interaction between GDF9 and BMP15 homodimers or from a GDF9/BMP15 heterodimer. In vitro, rGDF9/rBMP15 heterodimer has shown a potent activation of the SMAD2/3 pathway in mouse granulosa cells [38]; both SMAD2/3 and SMAD1/5/8 pathways in a human granulosa cell line (COV434) [8]; and better-supporting embryo development after IVM in the mouse and porcine models [7, 8]. Nonetheless, the unchanged concentration of the native GDF9/BMP15 heterodimer between GV and MII oocytes detected in the present study indicates the heterodimer is not regulated or affected during human IVM.

The concentration of GDF9 in spent media was approximately 10-times higher than BMP15, which corroborates recent measurements by our group in follicular fluid from human small antral follicles [39]. This 10-times excess GDF9 in relation to BMP15 indicates that the vast majority of GDF9 are not bound in a heterodimer complex and may have a role on its own during folliculogenesis.

Interestingly, similar GDF9 and BPM15 bands were detected in fresh oocytes, and in GV and MI/MII after IVM, indicating that these growth factors are produced both before and during maturation in vitro. The early oocyte specific expression of GDF9 and BMP15 was also detected in oocytes from COCs obtained from small antral follicles using immunofluorescence analysis, illustrating that GDF9 and BMP15 are expressed already in immature oocytes long before selection occurs.

GDF9 and BMP15 bind to receptors on the surrounding cumulus cells [9]. We hypothesize that the reduced concentrations of GDF9 and BMP15 detected in the spent media from MII oocytes are the results of higher consumption of GDF9 and BMP15 by their corresponding cumulus cells during IVM. Whether this reduction in GDF9 and BMP15 during IVM can be related to oocyte quality has to await fertilization studies. The significantly upregulated expressions of both the common receptor *BMPR2* and the downstream genes *SMAD3*, and *SMAD5* in cumulus cells from MII oocytes, indicate increased activity in these two signaling pathways, supporting this hypothesis. Apparently, both the GDF9-SMAD2/3 and the BMP15-SMAD1/5/8 axes are equally essential for maintaining ovarian function in mono-ovulatory species, since sheep homozygous for inactivating mutations in either GDF9 or BMP15 genes are infertile [20]. Another study including IVF patients also described a tendency for serum concentrations of GDF9 and BMP15 to decline after the LH surge [32]. This was, however, not significant due to patient effects and low detection efficiency. On the contrary, a relatively small study reports a positive correlation between GDF9 concentration in follicular fluid and oocyte maturation during IVF in a total of 6 fluid samples [13]. Nevertheless, the in vivo concentrations of GDF9 and BMP15 in follicular fluid from pre-ovulatory follicles are cumulative and do not represent solely the timeframe where the oocytes resumed meiosis. All COCs included in our study were carefully washed three times before IVM to make sure that we measured growth factors exclusively produced during the 42-h IVM period.

The present study has some limitations. Only the canonical GDF9 and BMP15 SMAD pathways were evaluated by gene expression. Evidence from rat granulosa cell cultures has demonstrated that human rGDF9 and rBMP15 may also activate other non-SMAD signaling pathways, such as the nuclear factor-_Κ_B (NF-_Κ_B) and the c-Jun N-terminal kinase (JNK) signaling pathways [40]. Measuring the expression of more genes from these non-SMAD pathways would strengthen the study. However, the amount of mRNA that can be extracted from a single COC to perform RT-qPCR is very limited. The timing of when the first polar body was released during IVM was not recorded. Further experiments are needed to evaluate the effect of the duration of MII arrest on the GDF9/BMP15 signaling during IVM.

The clinical relevance of this study would increase if the MII oocytes were fertilized and the embryo development evaluated, but the Danish authorities do not allow IVM in a clinical setting since the procedure is still considered experimental.

## Conclusions

For the first time, the in vivo forms of GDF9 and BMP15 in human oocytes have been determined with a Mw of 47 and 43 KDa, respectively. Further, lower GDF9 and BMP15 concentrations were detected in spent media from MII oocytes after IVM compared to GV oocytes, suggesting that these growth factors were used by the surrounding cumulus cells during oocyte meiotic resumption, while the GDF9/BMP15 heterodimer seemed to be of less importance. Moreover, *BMPR2*, *SMAD3*, and *SMAD5* were significantly upregulated in MII oocytes, indicating that both GDF9 and BMP15 pathways were active during oocyte meiotic resumption. These data suggest that the driving mechanisms for oocyte nuclear maturation may involve both GDF9 and BMP15 homodimers, with a huge surplus of GDF9 as compared to BMP15.

## Supplementary Information

**Additional file 1:** **Table S1.** Type of sample collected, and method used for each patient

**Additional file 2:** **Table S2.** Analytical characteristics of the ELISAs

**Additional file 3.** GDF9 and BMP15 in immature oocytes. Uncropped western blot membranes.

**Additional file 4.** GDF9 and BMP15 in GV and MI/MII oocytes after IVM. Uncropped western blot membranes.

## Data Availability

The datasets used and/or analysed during the current study are available from the corresponding author on reasonable request.

## Abbreviations

OTC: Ovarian tissue cryopreservation
IVM: In vitro maturation
COCs: Cumulus-oocyte complexes
L-COC: COC with large cumulus mass
S-COC: COC with small cumulus mass
NOs: Naked oocytes
GV: Germinal vesicle
MI: Metaphase I
MII: Metaphase II
DEG: Degenerated
rLH: Human recombinant luteinizing hormone
rFSH: Human recombinant follicle stimulating hormone
TGF-β: Transforming growth factor-beta
GDF9: Growth and differentiation factor 9
BMP15: Bone morphogenetic protein 15
BMPR2: BMP receptor type II
ALK: Activin receptor-like kinase
ACTB: Beta-actin
ELISA: Enzyme-linked immunosorbent
Mw: Molecular weight
RT-qPCR: Quantitative real-time PCR
