# Supplementary material for: Concentrations of oocyte secreted GDF9 and BMP15 decrease with MII transition during human IVM
Source: Reprod Biol Endocrinol. 2022 Aug 19;20:126. doi: 10.1186/s12958-022-01000-6 (PMC9389727; doi:10.1186/s12958-022-01000-6)
Supplement: Supplementary file 1 — Additional file 1: Table S1. Type of sample collected, and method used for each patient [file 12958_2022_1000_MOESM1_ESM.rtf]

				
Patient	Sample type	RT-qPCR	ELISA	Western blot	IF 	
1	Cumulus cells / Spent media 	*	*			
2	Cumulus cells / Spent media 	*	*			
3	Cumulus cells / Spent media	*	*			
4	Spent media		*			
5	Cumulus cells / Spent media	*	*			
6	Spent media		*			
7	Spent media		*			
8	Cumulus cells / Spent media	*	*			
9	Spent media		*			
10	Cumulus cells / Spent media	*	*			
11	Cumulus cells / Spent media	*	*			
12	Spent media		*			
13	Cumulus cells / Spent media	*	*			
14	Cumulus cells	*				
15	Cumulus cells	*				
16	Cumulus cells	*				
17	Cumulus cells	*				
18	Cumulus cells	*				
19	Cumulus cells	*				
20
…
58
59
60	Oocytes
Oocytes
Oocytes 
Cumulus-oocyte complex
Small antral follicle			*
*
*	


*
*	
Table S1 Type of sample collected, and method used for each patient


RT-qPCR: real-time quantitative PCR; IF: immunofluorescence. Cumulus cells (n = 72): Fresh GV (n = 24), GV after IVM (n = 21), and MII after IVM (n = 27). Spent media (n = 94): GV (n = 43) and MII (n = 51). Oocytes for western blot (n = 242): Fresh (n = 84) and after IVM (n = 158).  
