# Supplementary material for: Concentrations of oocyte secreted GDF9 and BMP15 decrease with MII transition during human IVM
Source: Reprod Biol Endocrinol. 2022 Aug 19;20:126. doi: 10.1186/s12958-022-01000-6 (PMC9389727; doi:10.1186/s12958-022-01000-6)
Supplement: Supplementary file 2 — Additional file 2: Table S2. Analytical characteristics of the ELISAs [file 12958_2022_1000_MOESM2_ESM.docx]

**Table S2** Analytical characteristics of the ELISAs

| **ELISA**  **Reagents** | **Antibody Binding Region** | **Dynamic Range** | **LoD** | **Imprecision**  **% CV @**  **(Measured Concentration)** | **Cross Reactant spiked at 10x of highest calibrator in Analyte free matrix**  **(Detectability)** | |
| --- | --- | --- | --- | --- | --- | --- |
| GDF-9  AL-176 | Mature GDF-9 Capture, Mature GDF-9 Detection | 48-5800 pg/mL | 3.0 pg/mL | 3.6 % (492.2 pg/mL)  2.1% (1335.0 pg/mL) | | GDF-9 (D), GDF-9:BMP-15 hetero dimer (D), BMP-15 (ND) |
| BMP15  AL-179 | Pro BMP-15 Capture, Mature BMP-15 Detection | 60-6700 pg/mL | 29 pg/mL | 3.3 % (480.5 pg/mL)  3.4% (1649.9 pg/mL) | | BMP-15 (D), GDF-9:BMP-15 hetero dimer (ND), GDF-9 (ND) |
| GDF9/BMP15-  Complex AL-181 | Mature GDF-9 Capture, Mature BMP-15 Detection | 30-5200 AU/mL | 8.7 AU/mL | 3.3 % (528.1 AU/mL)  5.6% (1569.0 AU/mL) | | GDF-9:BMP-15 hetero dimer (D)  BMP-15 (D), GDF-9 (ND) |

AU = Arbitrary Units, LoD = Limit of detection, D = Detectable, ND = Non-Detectable (< LoD). The cross reactants used in the analysis are commercially available unless otherwise specified. Inhibin A (AI035, AnshLabs), Inhibin B (BI043, AnshLabs), Activin A (338-AC/CF, R&D System), Activin B (659-AB/CF), Activin AB (1066-AB/CF), FSH (F4021, Sigma), TSH (T9265, Sigma), LH (L6420, Sigma), Estradiol (PHR1353, Sigma), FSTL-3 ( BF017, AnshLabs), FST-315 (4889-FN, R&D System), Mature AMH (1737-MS/CF, R&D System ), Pro+Mature AMH (BA047, AnshLabs), GDF-9 ( BG016, AnshLabs), BMP-15 (BB028, AnshLabs), GDF-9:BMP-15 heterodimer (BB023, AnshLabs).
