# Supplementary figures and images for: Concentrations of oocyte secreted GDF9 and BMP15 decrease with MII transition during human IVM
Source: Reprod Biol Endocrinol. 2022 Aug 19;20:126. doi: 10.1186/s12958-022-01000-6 (PMC9389727; doi:10.1186/s12958-022-01000-6)

**A Additional file 3. Western blots – uncropped membranes**

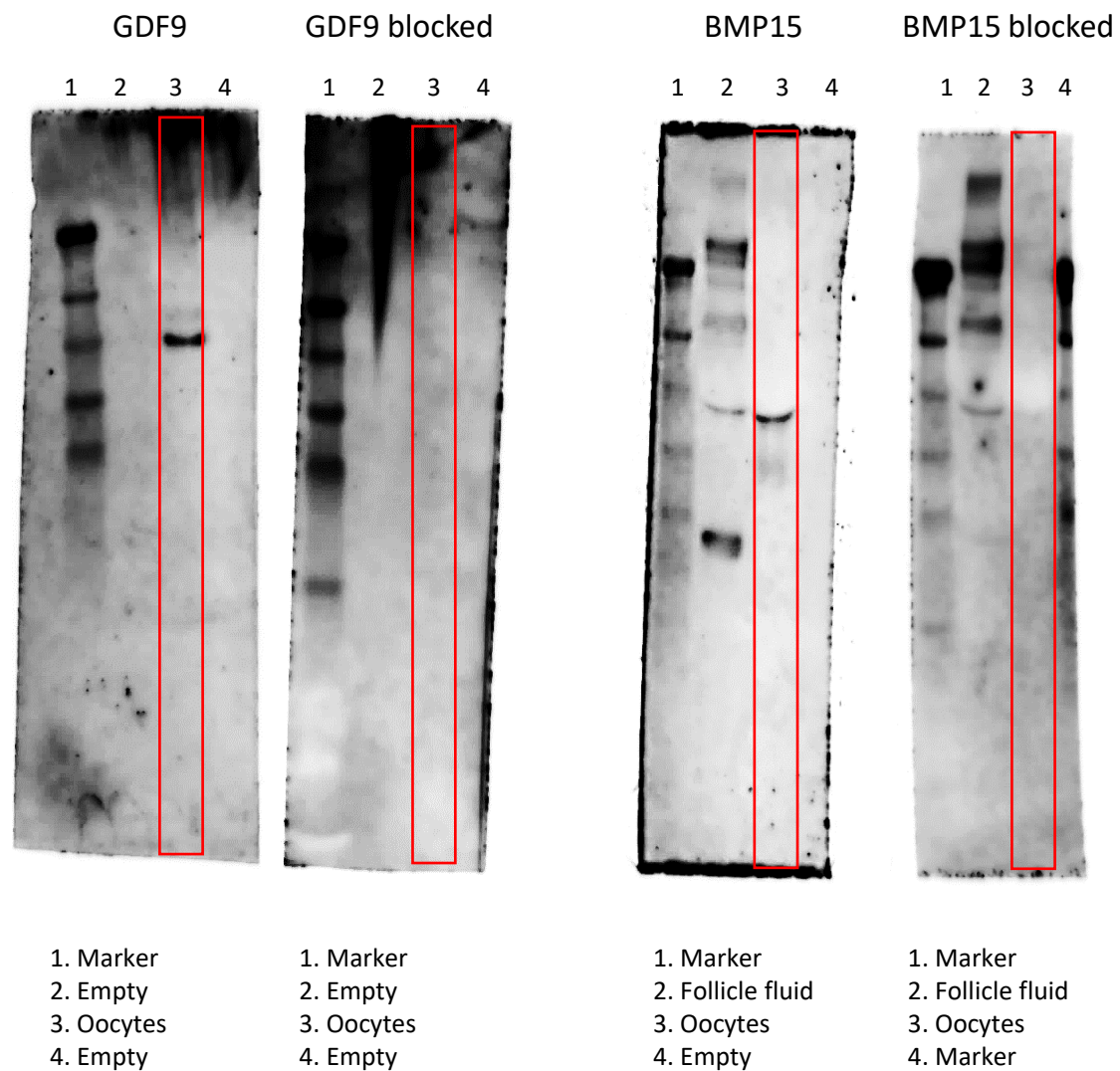

Supplement: Supplementary file 3 — Additional file 3. GDF9 and BMP15 in immature oocytes. Uncropped western blot membranes. [file 12958_2022_1000_MOESM3_ESM.pdf]
