# Supplementary material for: Concentrations of oocyte secreted GDF9 and BMP15 decrease with MII transition during human IVM
Source: Reprod Biol Endocrinol. 2022 Aug 19;20:126. doi: 10.1186/s12958-022-01000-6 (PMC9389727; doi:10.1186/s12958-022-01000-6)
Supplement: Supplementary file 4 — Additional file 4. GDF9 and BMP15 in GV and MI/MII oocytes after IVM. Uncropped western blot membranes. [file 12958_2022_1000_MOESM4_ESM.pdf]

B **Additional file 4.** Western blots – uncropped membranes

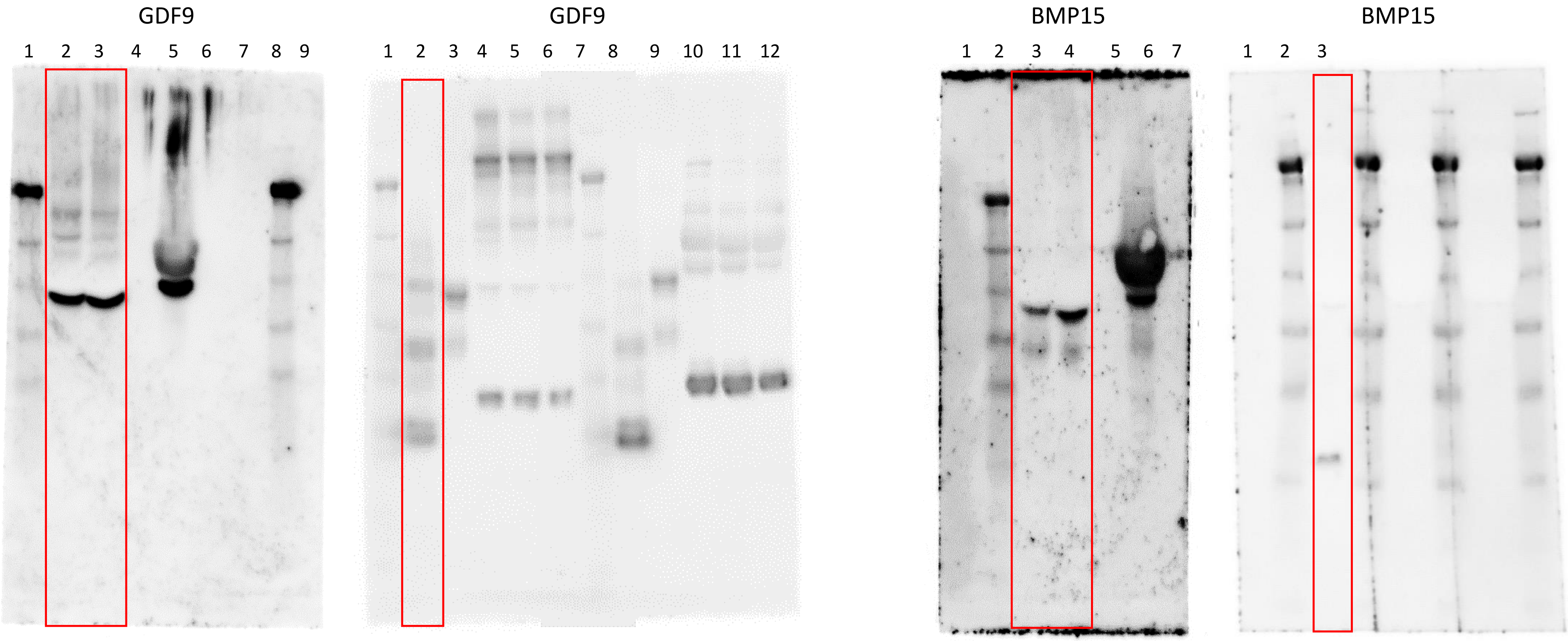

GDF9:

1. Marker
2. GV oocytes
3. MI/MII oocytes
4. Empty
5. MI/MII oocytes reduced
6. Empty
7. GDF standard
8. Marker
9. Empty

GDF9:

1. Marker
2. GDF9 standard
3. Oocytes
4. Follicle fluid
5. Follicle fluid
6. Pooled follicle fluid
7. Marker
8. GDF9 standard
9. Oocytes
10. Follicle fluid
11. Follicle fluid
12. Pooled follicle fluids

BMP15:

1. Empty
2. Marker
3. GV oocytes
4. MI/MII oocytes
5. Empty
6 . MI/MII oocytes reduced
7. Empty

BMP15:

1. Empty
2. Marker
3. BMP15 standard
